# Supplementary material for: Estimating affective polarization on a social network
Source: PLoS One. 2025 Sep 24;20(9):e0328210. doi: 10.1371/journal.pone.0328210 (PMC12459850; doi:10.1371/journal.pone.0328210)
Supplement: S1 File — Document including supporting analyses, figures, tables, and references. (PDF) [file pone.0328210.s001.pdf]

Supplementary Materials for “Estimating affective polarization on a social network”

Marilena Hohmann[1*], Michele Coscia[2]

1 Copenhagen Center for Social Data Science, University of Copenhagen, Copenhagen, Denmark

2 CS Department, IT University of Copenhagen, Copenhagen, Denmark

* marilena.hohmann@sodas.ku.dk

1 Alternative Measures

1.1 Average Sentiment

The average sentiment score $\mu_{o,y}$ compares the sentiment levels between people with similar opinions and those with opposing views. In the experiments presented in the main article, we introduce 0 as a threshold to split the nodes into a blue ($o_i \leq 0$) and a red group ($o_i > 0$). We calculate the average hostility $\mu_{o,y}$ *(Like-minded)* for all like-minded node pairs, that is, for all edges connecting nodes of the same color. Similarly, we calculate the average hostility $\mu_{o,y}$ *(Cross-cutting)* for all disagreeing node pairs, i.e., all edges connecting a blue and a red node.

1.2 Pearson Correlation Coefficient

The Pearson correlation captures the linear relationship between two variables x and y. In the case of affective polarization, the correlation between disagreement and hostility is indicative of in-group versus out-group hostility. The Pearson correlation coefficient $\rho_{o,y}$ is defined as follows:

$$\rho_{o,y} = \frac{\sum\limits_{(i,j)\in E} (x_{i,j} - \bar{x})(y_{i,j} - \bar{y})}{\sigma_x \sigma_y}$$

where $x_{i,j} = |o_i - o_j|$ are the disagreement values for each connected node pair in G, $y_{i,j}$ are the hostility values for each connected node pair in G, $\bar{x}$ and $\bar{y}$ are the mean values of the vectors x and y, and σ_x as well as σ_y are the standard deviations of x and y.

1.3 Earth Mover's Distance $EMD_{o,y,G}$

$EMD_{o,y,G}$ relies on the Earth Mover's Distance, a distance measure between two distributions [1], and the network measure Krackhardt's E/I index [2]. The premise of this measure is that the nodes in the network can be divided into two stance groups: k and k'; e.g., climate change believers and disbelievers in the case of [3]. Similar to the average sentiment scores $\mu_{x,y}$, we use 0 as a threshold to split the nodes into a blue ($o_i \leq 0$) and a red group ($o_i > 0$). Next, the weight of each edge w_{ij} is determined by the hostility of the interaction. In particular, w_{ij}^+ denotes all *non-hostile* edge weights, and w_{ij}^- denotes all *hostile* edge weights.[1]

The measure consists of two parts: the valence (sign) of $EMD_{o,y,G}$ and its magnitude, which are determined separately. Moreover, the measure is calculated for each stance group k separately.

1.3.1 EMD valence

To retrieve the sign of the score, the E/I index for the subgraph containing all non-hostile interactions G^+ is calculated as follows:

$$P_k^+ = \frac{E_k^+ - I_k^+}{E_k^+ + I_k^+}$$

where E_k^+ is the sum of all non-hostile *out-group* edges and I_k^+ is the sum of all non-hostile *in-group* edges. Similarly, the E/I index for the subgraph of all hostile interactions G^- is calculated as:

$$P_k^- = \frac{E_k^- - I_k^-}{E_k^- + I_k^-}$$

[1] The authors of [3] rely on a sentiment analysis to estimate sentiment values between ± 1 as an indicator for hostility. In their measure, they consider positive (> 0) and negative (< 0) interactions. Since the hostility values we generate in the experiments are in $[0, 1]$, we change the measure to non-hostile (≤ 0.5) and hostile interactions (> 0.5).

where E_k^- is the sum of all hostile *out-group* edges and I_k^- is the sum of all hostile *in-group* edges. The sign of the measure is then determined by:

$$P_k = \frac{P_k^- - P_k^+}{2}$$

If P_k results in a positive value, then the out-group interactions are disproportionally hostile, and affective polarization is high, while values close to 0 indicate low levels of affective polarization. Lastly, negative values of P_k indicate that the in-group interactions are especially hostile.

1.3.2 EMD Magnitude

To determine how affectively polarized a network is, the measure considers the distribution of out-group hostility u_k and the distribution of in-group hostility v_k. Next, it calculates the Earth Mover's Distance to determine the difference between the distributions. Finally, $EMD_{o,y,G}$ is defined as:

$$EMD_{o,y,G} \begin{cases} -\int_{-\infty}^{+\infty} |U_k - V_k| & : P_k < 0 \\ \int_{-\infty}^{+\infty} |U_k - V_k| & : P_k \geq 0 \end{cases}$$

where U_k and V_k are the cumulative distribution functions of u_k and v_k respectively. Intuitively, the Earth Mover's Distance captures how different the in-group hostility values are compared to the out-group hostility values. Moreover, P_k determines the sign of the final measure: if $P_k \geq 0$, then the measure has a positive sign, which indicates that affective polarization is high. Conversely, if $P_k < 0$, the measure has a negative sign, indicating more in-group than out-group hostility.

A few remarks about this measure should be noted. First, as shown in the main article, it does not appropriately account for the two components of affective polarization. Second, the measure assumes that two groups of individuals can always be clearly distinguished in the network, which proves difficult for cases where ideological leaning is modeled as a continuous variable, for instance, between ± 1. We solve this problem in the experiments by enforcing a split at 0. Consequently, the measure is more coarse since it treats nodes with an opinion score smaller (larger) than but close to 0,

the same as nodes with opinions close to the extreme -1 $(+1)$.

1.4 Structural Alignment Index $SAI_{o,y,G}$

The Structural Alignment Index $SAI_{o,y,G}$ proposed in [4] is a measure of frustration in signed graphs. To ensure that $SAI_{o,y,G}$ is compatible with the setup of our synthetic experiments, we make some adjustments to the measure and the experiment data.

The Structural Alignment Index works with a signed graph $G = (V, E, \sigma)$, where V denotes the set of nodes, E is the set of edges, and the mapping σ assigns a sign to each edge $E \to \{+, -\}$. We need to translate the hostility values into edge signs to use this measure in the synthetic experiments. To achieve this, we introduce a threshold: all hostility values $y_{i,j} > 0.5$ are converted into a negative sign, and all hostility values $y_{i,j} \leq 0.5$ are converted into a positive sign. This corresponds to the notion that a high hostility value indicates a negative interaction, while a low hostility value indicates a positive interaction.

Next, the authors of [4] determine a node partition that minimizes the number of frustrated edges in the network. In contrast to their approach, we divide the nodes into a blue group ($o_i \leq 0$) and a red group ($o_i > 0$) based on the available opinion values in the experiments. This division into two groups aligns with the finding in [4] that the optimal partition in their examples consistently comprised two groups. Importantly, this partition might not necessarily minimize the count of frustrated edges, but it is well-suited for the type of data we are working with.

Given the {red, blue} partition, we calculate the frustration index L^*_G. The frustration index is defined as the count of edges that require removal (or change of sign) for the network to achieve balance. In a signed graph, frustrated edges correspond to either positive out-group edges or negative in-group edges, and we count these to determine L^*_G.

In the next step, the authors introduce a null model to normalize their measure. In the null model, the edge signs are randomly shuffled while maintaining the network structure and partition. The frustration index $L_{\widetilde{G}}$ of the null model is calculated as the mean frustration across several shuffles. For the results presented in the main paper, we opt for ten repetitions.

Finally, the Structural Alignment Index $SAI_{o,y,G}$ is defined as:

$$SAI_{o,y,G} = 1 - \frac{L^*_G}{L_{\widetilde{G}}}$$

1.5 Polarized Embeddings for Signed Networks $POLE_{y,G}$

$POLE_{y,G}$ (Polarized Embeddings for Signed Networks) is a method developed for the purpose of signed link prediction. In addition to link prediction, the authors define a polarization measure on a signed graph, which we focus on here [5]. Like the method described in the previous section, $POLE_{y,G}$ requires a signed graph $G = (V, E, \sigma)$ with V as the set of nodes, E as the set of edges, and a mapping σ assigning signs to edges $E \to \{+, -\}$. As before, we need to turn the hostility values into edge signs: all hostility values $y_{i,j} > 0.5$ are converted into a negative sign, and all hostility values $y_{i,j} \leq 0.5$ are converted into a positive sign.

As opposed to the previous measures, $POLE_{y,G}$ disregards any information about the node opinions or node partitions. Instead, it exclusively focuses on the graph structure and edge signs. In particular, $POLE_{y,G}$ compares transition probabilities of random walks on the unsigned and the signed graph.

For the unsigned graph, the matrix $\mathbf{U}$ denotes the random walk transition probabilities. For instance, $\mathbf{U}_{i,j}$ is the transition probability between nodes i and j. For the signed graph, the authors add signs to the transition probabilities. The inferred sign between nodes i and j is based on the product of the signs of all edges encountered during a random walk. For example, if the random walk between node i and node j first crosses an edge with a negative sign $(-)$, followed by an edge with a positive sign $(+)$, the inferred sign is calculated as $-1 \times 1 = -1$. In other words, the inferred sign for the random walk between i and j is negative $(-)$. These signed transitions are represented in a matrix $\mathbf{S}$.

Next, the analysis involves the calculation of a node-level polarization measure. For node i, this measure is defined as the Pearson correlation between the signed and unsigned transition values [5]:

$$pol(i) = corr(\mathbf{U}_{i,*}, \mathbf{S}_{i,*})$$

Lastly, the network-level polarization measure $POLE_{y,G}$ is the average across the node-level polarization values [5]:

$$POLE_{y,G} = \frac{\sum_i pol(i)}{|V|}$$

2 Sensitivity to the strength of opinions

We perform an additional synthetic experiment testing the sensitivity of the different measures to the strength of opinions. For this experiment, we generate a random $G_{n,m}$ graph with $n = 50$ nodes and $m = 610$ edges. Then, we generate a vector o^* containing 25 equispaced opinion values $o_i \in [c - 0.1, c]$. The parameter c determines the strength of the opinions: (a) $c = 0.2$, (b) $c = 0.4$, (c) $d = 0.6$, (d) $e = 0.8$, (e) $c = 1.0$. The final opinion vector is $o = (o^*, -o^*)$.

To generate the hostility values, we consider the opinion vector o and respective disagreement vector x in the network shown in Fig 1(e). Based on x, we generate a positive disagreement–hostility correlation by specifying the hostility parameter $a = 0.25$ (see Materials and Methods in the main article). The hostility values $y_{i,j}$ are fixed across all examples in Fig 1.

While the number of disagreeing node pairs is the same across all examples, the nodes in (a) all have moderate opinions close to 0, while the opinions in (e) are close to the extremes of the opinion spectrum. Since the opinions get uniformly more extreme throughout the examples in Fig 1, we expect polarization to increase from (a) to (e). Our affective polarization measure $\alpha_{o,y,G}$ confirms this expectation as its values increase from (a) to (e).

On the contrary, the other measures we test cannot capture changes in the strength of opinions. The Pearson correlation coefficient $\rho_{o,y}$ shows a slight difference between (a) and (b), but it cannot distinguish between any of the remaining networks in Fig 1. $\mu_{x,y}$, $EMD_{o,y,G}$, $SAI_{o,y,G}$, and $POLE_{x,G}$ do not capture any changes in the opinion distribution, as the values are the same for all the networks considered in this experiment.

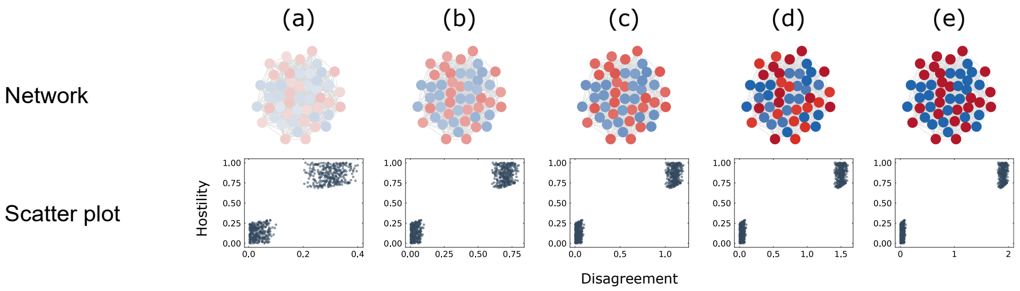

	(a)	(b)	(c)	(d)	(e)
Affective polarization $\alpha_{o,y,G}$	**(0.17, 0.17)**	**(0.39, 0.39)**	**(0.62, 0.62)**	**(0.84, 0.84)**	**(1.06, 1.07)**
Pearson correlation $\rho_{o,y}$	(0.95, 0.95)	(0.97, 0.97)	(0.97, 0.97)	(0.97, 0.98)	(0.97, 0.98)
Avg $\mu_{o,y}$ (Like-minded)	(0.13, 0.14)	(0.13, 0.14)	(0.13, 0.14)	(0.13, 0.14)	(0.13, 0.14)
Avg $\mu_{o,y}$ (Cross-cutting)	(0.85, 0.85)	(0.85, 0.85)	(0.85, 0.85)	(0.85, 0.85)	(0.85, 0.85)
$EMD_{o,y,G}$ (Blue)	(0.71, 0.72)	(0.71, 0.72)	(0.71, 0.72)	(0.71, 0.72)	(0.71, 0.72)
$EMD_{o,y,G}$ (Red)	(0.71, 0.72)	(0.71, 0.72)	(0.71, 0.72)	(0.71, 0.72)	(0.71, 0.72)
$SAI_{o,y,G}$	(1.00, 1.00)	(1.00, 1.00)	(1.00, 1.00)	(1.00, 1.00)	(1.00, 1.00)
$POLE_{y,G}$	(0.07, 0.09)	(0.07, 0.09)	(0.07, 0.09)	(0.07, 0.09)	(0.07, 0.09)

Fig 1. Sensitivity to the strength of opinions. From top to bottom: network where the node color reflects opinion, from -1 (blue), passing 0 (white), to $+1$ (red); scatter plot with disagreement on the x-axis and hostility on the y-axis; values of $\alpha_{o,y,G}$, $\rho_{o,y}$, $\mu_{o,y}$, $EMD_{o,y,G}$, $SAI_{o,y,G}$, and $POLE_{y,G}$. The results (in parentheses) denote the lower and upper bound of the 95% confidence interval across 100 repetitions of the experiment.

3 Twitter Data

3.1 English Language Detection

To detect English-language tweets, we choose a pre-trained version of the fastText language model [6, 7]. This model returns a set of detected languages and their classification probabilities. Fig 2 shows the distribution of these probability scores for detecting English-language tweets in the Twitter sample. We choose a relatively high threshold of 0.8, above which we consider tweets to be English-speaking to ensure that further analyses, particularly the toxicity classification, are not distorted by non-English tweets.

3.2 Keyword List: COVID-19 Restrictions

We filter the initial dataset to obtain tweets referencing COVID-19 restrictions specifically. Since defining a comprehensive list of keywords is challenging, we train a word2vec model on the tweet corpus. This approach allows us to explore the corpus and identify Twitter-specific terms mentioned in the tweets. We manually collect a list of

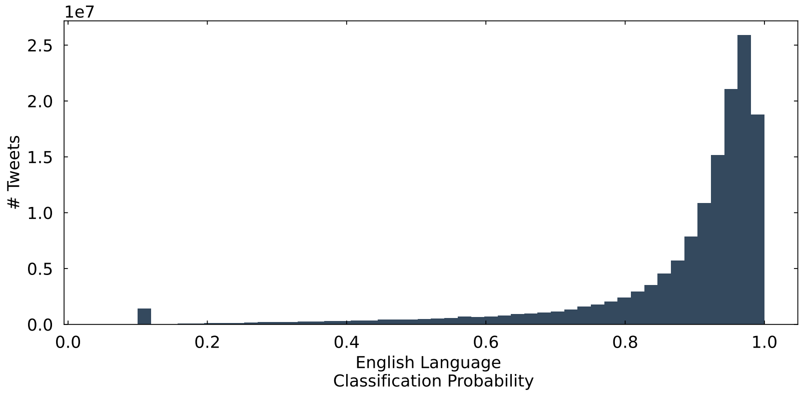

Fig 2. Distribution of the language detection probabilities. Number of tweets (y-axis) with a given English likelihood score (x-axis).

keywords on COVID-19 restrictions and extend this list with synonyms, hashtags, and other relevant terms suggested by the trained model. We also curate a list of COVID-19-skeptical expressions. Since these pandemic-skeptic users do not seem to mention terms that specifically discuss the restrictions but instead reject the idea of a pandemic in general, the COVID-19-skeptical keywords are broad. The final list of stemmed keywords and hashtags is shown below, including three emojis that were frequently used by users discussing COVID-19 restrictions (Fig 3).

Keywords on COVID-19 restrictions. *#alonetogether, #asktheexperts, , #avoidcrowds, #coveryourcough, #coveryourface, #dontbeacovidiot, #fightcovid, #flattenthecurve, #keepingyousafe, #quaranteam, #quaratinelife, #shelterinplace, #sixfeetapart, #stayingsafe, #staysafeeveryone, #staysafestayhealthy, #stoppingthespread, #stopthespreadofcovid, #togetherapart, abid, adher, antigen, ban, clorox, clos, complianc, contact, curb, curfew, decontamin, deterg, disinfect, dispens, distanc, guidanc, guidelin, home, hygein, hygien, isol, lockdown, lysol, mask, measur, minim, mitig, pcr, plexiglass, precaut, prevent, procedur, protect, protocol, quarantin, quarentin, recommend, requir, respons, restrict, result, sanat, sanit, school, sheild, shield, test, touchless, touchpoint, trace, wash, wear*

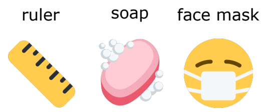

Fig 3. Emojis used to match tweets discussing Covid-19 restrictions.

Covid-19-skeptical keywords. *#americawakeup, #covidlies, #covidscamdemic,*

#falseflag, #fauxnews, #medialies, #mediascum, #msmistheenemyofthepeople, #nonewnormal, #openamericanow, #plandemic, #plannedemic, #qanons, #scummedia, #sheeple, #thegreatawakening, #wearethenewsnow, #wethepeople, #wwgwga, brainwash, cherrypick, chinaviru, conspirac, debunk, dishonest, fake, fascism, fauci, fearmong, fraudci, hoax, honkler, hype, hyster, kungflu, lamestream, mouthpiec, parrot, propagan, puppet, scaremong, strawman

3.3 User Opinion Scores

3.3.1 Distribution

As the Materials and Methods section in the main article outlines, we examine the users' retweeting behavior to infer their opinion scores. To match the DW-NOMINATE scores of each politician in the 118th US Congress (2019–2021) to a Twitter account, we use a comprehensive list provided in [8].[2] Moreover, we retrieve ideological leaning scores for news media outlets from mediabiasfactcheck.com (MBFC). This website scores the leaning of news outlets, and this data has been used for similar purposes in previous studies [9, 10]. We collect all names of news outlets on the MBFC website and subsequently look up the associated Twitter accounts. Fig 4 shows the distribution of political leaning scores among the set of political and media accounts (on the left) and the opinion distribution for all users that retweeted at least five posts by one or more of these accounts (on the right).

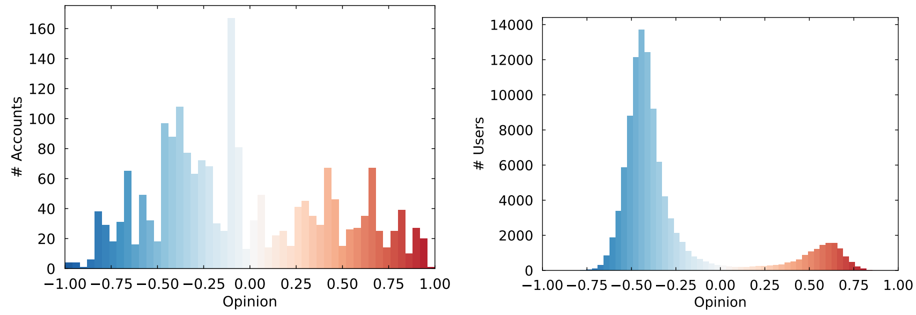

(a) Political and media accounts **(b)** Users

Fig 4. Opinion distribution. Number of accounts (y-axis) with a given opinion value (x-axis and bar color). (a) Distribution of political and media account scores; (b) distribution of user scores for users who retweeted at least five posts by political and/or media accounts.

[2]The data is available in the associated GitHub repository at https://github.com/sdmccabe/new-tweetscores.

3.3.2 Opinion Threshold

We choose a threshold of five retweets to ensure that the retweeting behavior indeed indicates a political preference. As Fig 5 shows, the shape of the distribution is the same regardless of the threshold chosen.

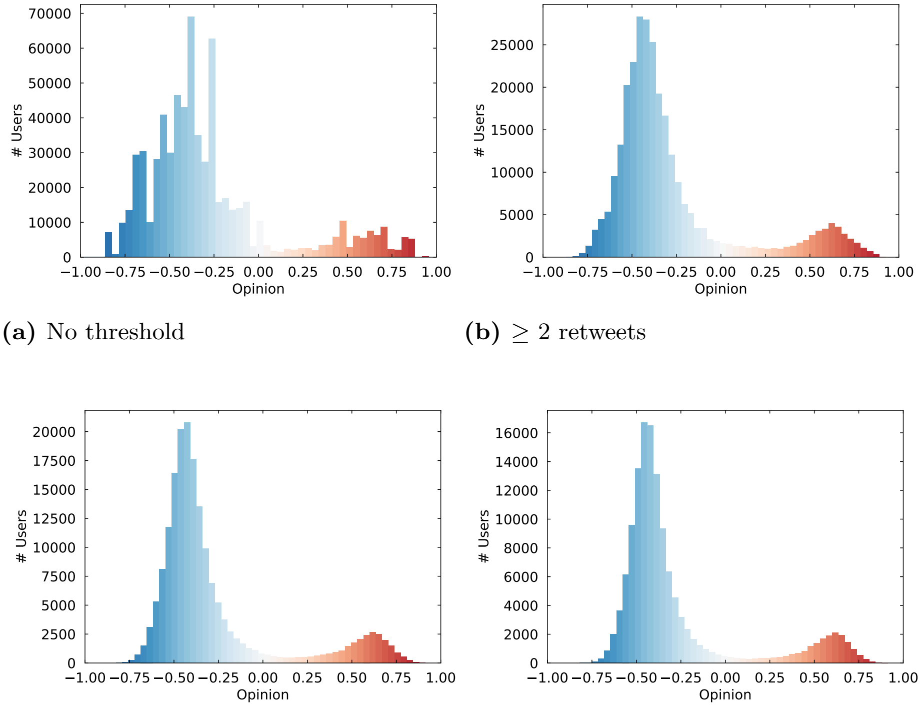

(a) No threshold (b) ≥ 2 retweets (c) ≥ 3 retweets (d) ≥ 4 retweets

Fig 5. Distribution of user opinion scores for different retweet thresholds. Same legend as Fig 4.

3.4 Toxicity Classification

3.4.1 Distribution

Fig 6 shows the distribution of classification probabilities returned by the toxicity classifier we use [11]. As expected, the toxicity probability is close to 0 for the vast majority of tweets. Approximately 9% of the tweets have a probability larger than 0.5, which serves as a threshold for identifying toxic tweets in our data analysis.

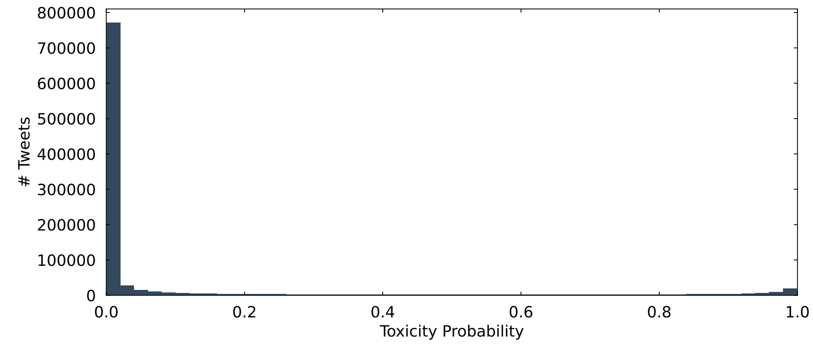

Fig 6. Distribution of probabilities for the toxicity classification. Number of tweets (y-axis) with a given toxicity score (x-axis).

3.4.2 Hostility Measurement

The Twitter interaction networks are undirected and unweighted. Since there can only be one edge between two individuals, we need to summarize the toxicity scores for multiple interactions. We do so by calculating the mean toxicity score for each node pair. Other approaches could have entailed summing all toxicity values or taking the maximum score instead of the average. Therefore, we calculate the affective polarization level for several toxicity thresholds and different approaches to summarizing interactions between two users. As Fig 7 shows, the results and their interpretation remain the same, regardless of the analytical choices.

We use toxic language to determine how hostile the user communication on Twitter is. However, other text classification methods might serve as an indicator of hostility. For instance, prior studies have used the sentiment of messages to investigate affective polarization on social media [3, 12–14]. As we argue in the main article, the sentiment score of a message often depends on the topic of discussion. In the case of COVID-19, users discuss somber topics such as death or disease, which should not be interpreted as a sign of out-group hostility.

Another alternative is offensive language classification. As an additional analysis, we use a RoBERTa-base model re-trained on approximately 58 million tweets and fine-tuned for detecting offensive language by [15]. Fig 8 shows the development of affective polarization in the Twitter data set when using offensiveness as an indicator of hostility.

Similar to the toxicity classification results, the offensiveness classifier returns a probability for each prediction. We show the results for three offensiveness probability

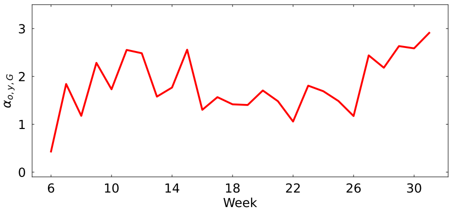

(a) Threshold 0.4; max edge attributes

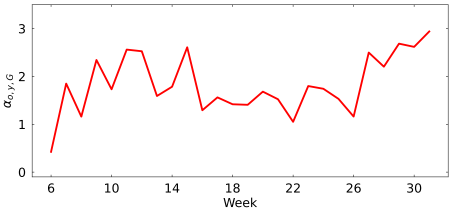

(b) Threshold 0.4; summed edge attributes

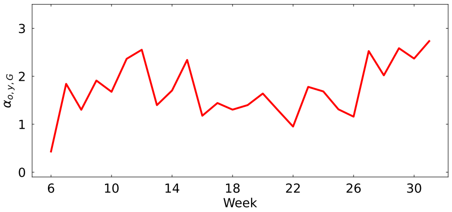

(c) Threshold 0.5; max edge attributes

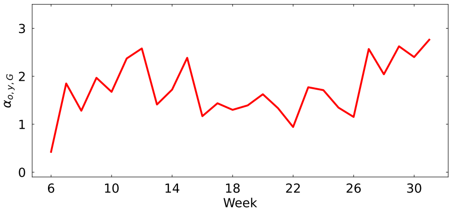

(d) Threshold 0.5, summed edge attributes

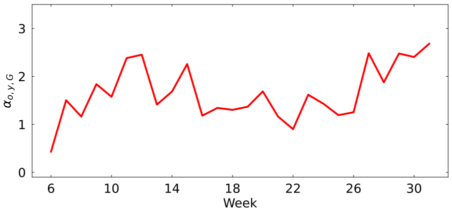

(e) Threshold 0.6; max edge attributes

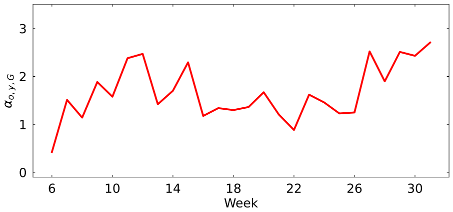

(f) Threshold 0.6; summed edge attributes

Fig 7. Results for different toxicity specifications. The rows show $\alpha_{o,y,G}$ for binary toxicity classes with 0.4 (first row), 0.5 (second row), and 0.6 (third row) as the threshold. Left: maximum toxicity chosen to summarize multiple edges between two individuals. Right: toxicity values are summed for node pairs with more than one interaction.

thresholds – 0.4, 0.5, and 0.6 – based on which we determine the offensiveness label of a message. We summarize multiple replies or mentions between two users by calculating the mean offensiveness score of all their interactions.

Fig 8 confirms that the conclusions drawn in the main article remain the same, irrespective of whether offensiveness or toxicity is used: in early February 2020, the level of affective polarization was relatively low, reflecting the limited engagement of users in the discourse concerning COVID-19 restrictions. As more users joined the debate in the spring and summer, we find high levels of affective polarization.

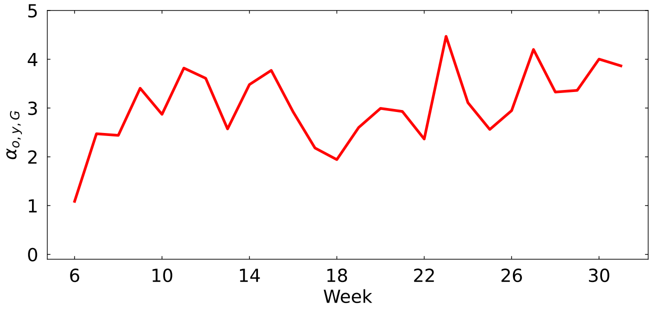

(a) Threshold 0.4, mean edge attributes

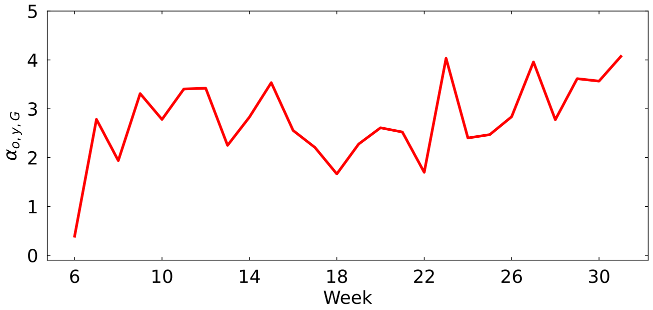

(b) Threshold 0.5, mean edge attributes

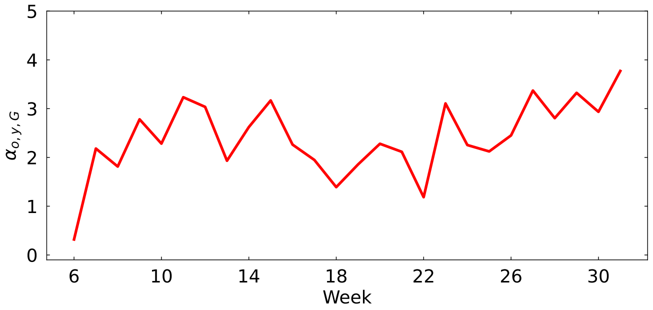

(c) Threshold 0.6, mean edge attributes

Fig 8. Results for different offensiveness specifications. The rows show $\alpha_{o,y,G}$ for binary offensiveness classes with 0.4 (first row), 0.5 (second row), and 0.6 (third row) as the threshold. To summarize multiple edges, we calculated the mean offensiveness score across all interactions.

3.5 Network Summary Statistics

Table 1 reports some summary statistics for the series of Twitter networks: the number of nodes n, the number of edges m, and the density D. Moreover, the table includes the average shortest path length l, and the modularity Q which we calculate based on a partition of nodes into a blue group ($o_i \leq 0$) and a red group ($o_i > 0$).

The summary statistics indicate that the networks are very sparse and do not exhibit a community structure. Moreover, Table 1 shows that there were only a few hundred users in February before considerably more users engaged in the Twitter debate in early March. As argued in the main article, this likely explains the low level of affective polarization in February 2020.

Week	n	m	D	l	Q
6	439	494	0.005	5.841	0.127
7	454	512	0.005	6.715	0.099
8	633	736	0.004	5.948	0.064
9	5418	8811	0.001	4.685	0.017
10	6057	10055	0.001	4.783	0.009
11	8162	15041	0.000	4.599	0.003
12	5563	8458	0.001	5.124	0.008
13	8070	13713	0.000	4.833	0.028
14	10339	17623	0.000	4.794	0.045
15	12257	20825	0.000	4.818	0.057
16	13926	24534	0.000	4.823	0.046
17	12681	21850	0.000	4.860	0.057
18	8709	14035	0.000	4.941	0.061
19	9059	14397	0.000	5.044	0.048
20	8527	13635	0.000	4.938	0.043
21	7947	12158	0.000	5.088	0.052
22	5996	8511	0.000	5.408	0.071
23	4499	6159	0.001	5.649	0.076
24	4876	6524	0.001	5.653	0.051
25	6274	8805	0.000	5.454	0.045
26	5629	8443	0.001	5.175	0.018
27	7700	11260	0.000	5.312	0.018
28	8740	13824	0.000	5.047	0.018
29	10001	16266	0.000	4.921	0.028
30	7845	12373	0.000	5.016	0.024
31	8353	12986	0.000	4.898	0.005

Table 1. Summary statistics for the Twitter networks: number of nodes n, number of edges m, density D, average shortest path length l, and modularity Q. To calculate Q, we partition the network into a community of blue nodes ($o_i \leq 0$) and a community of red nodes ($o_i > 0$).

References

1. Levina E, Bickel P. The Earth Mover's distance is the Mallows distance: Some insights from statistics. In: Proceedings Eighth IEEE International Conference on Computer Vision. ICCV 2001. vol. 2. IEEE; 2001. p. 251–256.

2. Krackhardt D, Stern RN. Informal networks and organizational crises: An experimental simulation. Social psychology quarterly. 1988; p. 123–140.

3. Tyagi A, Uyheng J, Carley KM. Heated conversations in a warming world: affective polarization in online climate change discourse follows real-world climate anomalies. Social Network Analysis and Mining. 2021;11(1).

4. Fraxanet E, Pellert M, Schweighofer S, Gómez V, Garcia D. Unpacking polarization: Antagonism and alignment in signed networks of online interaction. PNAS Nexus. 2024;3(12).

5. Huang Z, Silva A, Singh A. POLE: polarized embedding for signed networks. In: Proceedings of the Fifteenth ACM International Conference on Web Search and Data Mining. ACM; 2022. p. 390–400.

6. Joulin A, Grave E, Bojanowski P, Douze M, Jégou H, Mikolov T. FastText.Zip: compressing text classification models; 2016. Available from: https://arxiv.org/abs/1612.03651.

7. Joulin A, Grave E, Bojanowski P, Mikolov T. Bag of tricks for efficient text classification. In: Proceedings of the 15th Conference of the European Chapter of the Association for Computational Linguistics: Volume 2, Short Papers. Association for Computational Linguistics; 2017. p. 427–431.

8. McCabe S, Green J, Wan A, Lazer D. New tweetScores: or, did Donald Trump break tweetScores? Midwestern Political Science Assocation, Chicago, IL. 2022;.

9. Cinelli M, De Francisci Morales G, Galeazzi A, Quattrociocchi W, Starnini M. The echo chamber effect on social media. Proceedings of the National Academy of Sciences. 2021;118(9).

10. Hohmann M, Devriendt K, Coscia M. Quantifying ideological polarization on a network using generalized Euclidean distance. Science Advances. 2023;9(9).

11. Hanu L, Unitary team. Detoxify; 2020. Available from: https://github.com/unitaryai/detoxify.

12. Marchal N. “Be Nice or Leave Me Alone”: an intergroup perspective on affective polarization in online political discussions. Communication Research. 2022;49(3):376–398.

13. Yarchi M, Baden C, Kligler-Vilenchik N. Political polarization on the digital sphere: a cross-platform, over-time analysis of interactional, positional, and affective polarization on social media. Political Communication. 2021;38(1-2):98–139.

14. Mentzer K, Fallon K, Prichard J, Yates DJ. Measuring and unpacking affective polarization on Twitter: the role of party and gender in the 2018 Senate races. In: Proceedings of the 53rd Hawaii International Conference on System Sciences; 2020.

15. Barbieri F, Camacho-Collados J, Espinosa Anke L, Neves L. TweetEval: unified benchmark and comparative evaluation for tweet classification. In: Findings of the Association for Computational Linguistics: EMNLP 2020. Association for Computational Linguistics; 2020. p. 1644–1650.
