# Supplementary material for: Estimating affective polarization on a social network
Source: PLoS One. 2025 Sep 24;20(9):e0328210. doi: 10.1371/journal.pone.0328210 (PMC12459850; doi:10.1371/journal.pone.0328210)
Supplement: Attachment — Submitted filename: review_pone.pdf [file pone.0328210.s002.pdf]

This work introduces a novel method for measuring affective polarization, incorporating both affective and structural dimensions. By combining statistical correlation measures with a generalized Euclidean distance to assess ideological polarization, the authors derive a unique index to quantify affective polarization and apply it to both synthetic scenarios and Twitter data. Overall, the paper is well-written and provides sufficient detail about the proposed methodology.
Nevertheless, I have several concerns regarding the theoretical framework and the interpretation of the proposed measure.
From a theoretical perspective, the authors do not clearly articulate how they conceptualize affective polarization and how they relate it to ideological polarization. In the existing literature, there is no consensus on whether these two forms of polarization are distinct[1,2] or if ideological polarization is a key driver of partisan animosity[3,4]. The proposed measure combines two distinct terms: one representing the affective dimension and another based on the Euclidean distance within the network to quantify ideological polarization. However, it is unclear why a measure for affective polarization should account also for structural properties of the network/ ideological polarization, and not only rely on proxies measuring the attitude of users towards each others. Whether the two dimensions are treated as independent or correlated, the rationale behind multiplying these factors is not clear. This raises questions about the conceptual basis of the proposed measure and what it is actually able to quantify. The authors should clarify why a measure of affective polarization—defined as “the affective attitude toward like-minded and disagreeing others, concerned with in-group versus out-group dynamics”—should depend on a structural metric.

Additional Comments:
- The section titled "Methods" should be renamed, as it overlaps with the later "Materials and Methods" section.
It is unclear why the definition of the measure and the synthetic data analysis use Pearson correlation, whereas the other part of the analysis use Spearman correlation. If the correlation between hostility and disagreement is nonlinear (a finding I agree with), why is Pearson correlation used to define the measure?
The analysis of Twitter data is compelling; however, I suspect that the affective and structural dimensions of the measure are highly correlated. The authors should compare their measures with others and visualize the trends of the two components to provide additional insight.
The range of the proposed measure is not well-defined. This raises the question of whether the measure is suitable for comparing different networks or if it should be limited to tracking changes within the same network over time.

[1] Kligler-Vilenchik, N., Baden, C. & Yarchi, M. Interpretative Polarization across Platforms: How Political Disagreement Develops Over Time on Facebook, Twitter, and WhatsApp. Soc. Media Soc. 6, 205630512094439 (2020).

[2]Jost, J. T., Baldassarri, D. S. & Druckman, J. N. Cognitive–motivational mechanisms of political polarization in social-communicative contexts. Nat. Rev. Psychol. 1, 560–576 (2022).

[3] Fiorina, M. P., Abrams, S. A. & Pope, J. C. Polarization in the American Public: Misconceptions and Misreadings. J. Polit. 70, 556–560 (2008).

[4] Lelkes, Y. Mass Polarization: Manifestations and Measurements. Public Opin. Q. 80, 392–410 (2016).
