# Supplementary material for: Estimating affective polarization on a social network
Source: PLoS One. 2025 Sep 24;20(9):e0328210. doi: 10.1371/journal.pone.0328210 (PMC12459850; doi:10.1371/journal.pone.0328210)
Supplement: Attachment — Submitted filename: cover_letter_and_response.pdf [file pone.0328210.s003.pdf]

Estimating affective polarization on a social network

Marilena Hohmann[1*], Michele Coscia[2]

1 Copenhagen Center for Social Data Science, University of Copenhagen, Copenhagen, Denmark
2 CS Department, IT University of Copenhagen, Copenhagen, Denmark

* marilena.hohmann@sodas.ku.dk

To the editors of PLOS ONE,

This document outlines our point-by-point rebuttal to the reviewers. For each point raised, we indicate where and how we modified the original text, or argue why we decided not to.

Reviewer 1

1. (...) I don't think referring to the "structural" component of AP is particularly clear. Rather, I think the framing in terms of social distance offers more clarity. I would perhaps suggest reframing title / abstract to explicitly highlight this novel contribution of your work, e.g. "A social network measure of affective polarization which captures both hostility and social distance". You could also perhaps touch on why researchers may, or may not, want to include these different elements in their measure.

   - Following the reviewer's suggestion, we have revised the abstract and now explicitly refer to social distance. To improve clarity and consistency, we now refer to this component as the "social distance component" rather than the "structural component" throughout the paper.

2. You could be bolder in stating the benefits of your approach and possible useful applications.

   - We have adjusted part of the introduction to emphasize how our measure complements the existing methods in this area (see following point).

3. I would perhaps suggest not to refer to the survey-based approaches as having shortcomings (...) Rather I think your framing may be stronger by talking about your added value instead of the shortcomings of others. Also, what you call the structural component others may refer to as interactional polarization (see your Ref 11 and Yarchi et al. 2021 in Political Communication).

   - To address the first point, we have rewritten part of the introduction. Instead of emphasizing shortcomings, the paragraph now focuses on how our measure complements the survey-based approaches. Regarding the second point, we have included a reference to 'interactional polarization' research in the (renamed) 'Affective Polarization Measure' section.

4. (...) I would politely note that at least one of the papers referenced (Ref 11) is also using toxicity, not sentiment as you state.

- We have included [1] (the old Ref 11) in the paragraph that discusses sentiment vs. toxicity.

5. On page 5 you state: "This opinion homophily indicates high structural separation and, therefore, high affective polarization." I understand this argument, but there is plenty of data to suggest that hostile out-group interactions are not so rare (...). It is very dependent on interaction type (...). The measures of social distance typically used are quick high-stakes interactions (e.g., getting married, being neighbors) unlike social media interactions which are low stakes and are therefore more likely to include out-group interactions.

   - The (revised) Fig. 5 suggests that social distance may be a distinct driver of affective polarization in online networks, separate from affect itself: The two components are negatively correlated, i.e., as hostility decreases, structural separation and thus social distance increases.

     In general, it is important to note that our proposed measure is agnostic to the type of social interaction. While we apply it to Twitter data, the same method can also be used for offline networks, where connections represent high-stakes interactions, such as marriage or friendships. We agree with the reviewer's concern but view this as a limitation of the Twitter data set rather than the measure itself.

6. You should perhaps briefly note the assumptions underlying your measure's desire for a positive correlation between disagreement and hostility. If by disagreement you mean disagreement in terms of ideological identification that is fine, but that is not necessarily the same as disagreement on actual issues. See for example the papers by Liliana Mason (I Disrespectfully disagree) which discussed how, surprisingly, affective polarization often manifests as substantial hostility between partisan opponents even when those individuals largely *agree* on the details of policy.

   - As we discuss in the introduction, categorizing individuals into an in-group and an out-group overlooks some nuances in political orientation. To allow for greater flexibility, we use a correlational measure. In this framework, the only way to capture out-group hostility – a defining feature of affective polarization – is by implementing a positive correlation between disagreement and hostility.

     Similar to our previous response, we want to emphasize that our measure is agnostic to the type of disagreement considered. For our Twitter analysis, we focus on ideological disagreement, thereby avoiding the interpretational challenges the reviewer describes, where hostility arises despite agreement on policy issues. Generally speaking, it is up to the analyst to decide how to operationalize disagreement based on their data set.

7. In your AP measure, any particular reason you chose to equally weight structure and affect?

   - We choose not to assign more weight to either affect or structure because the political science literature lacks consensus on which aspect of polarization is more important. We therefore opted for an approach that treats both components equally.

8. The method validation section could be a little improved in terms of structure. You are using a lot of acronyms which some readers may not be familiar with.

You could take a little more time to introduce the other measures and note why they have been used in the past.

- We have restructured the method validation section. The alternative methods are now presented in a bulleted list, with explanations of how they work and are used.

9. Personally, I find Fig 2 a bit messy with all the different metrics included in one figure. I'd probably move most of the other metric results to the SI, but its up to you.

    - We understand the concern about the figure, but believe that Figures 2–4 present key results of the paper. Since the comparison with other metrics is essential to highlight the advantages of our proposed method, we have decided to keep the figures as they are.

10. Lines 274-275. When you talk about ideological labelling, remember to reference the methods section, otherwise we are confused how you have done this.

    - We have added a reference to the Materials and Methods section here.

11. In Figure 5, perhaps consider showing the individual components of the AP measure and then commenting on how the individual components compare to the combined measure.

    - We have updated Fig 5 to display our affective polarization measure alongside its separate components. We have also included a paragraph that discusses how the affective and social distance components evolve differently, providing a clearer explanation of how they each contribute to the overall level of affective polarization observed.

12. Lines 336 - 337: "These findings align with previous research indicating a consistent upward trend in affective polarization since the 1960s". Statements like this should be precise about exactly which country you are referring to. I note that AP has decreased in many countries! See Boxell & Gentzkow's NBER paper on AP across countries.

    - The sentence now specifically refers to increasing affective polarization in the United States.

Reviewer 2

1. It is unclear why a measure for affective polarization should account also for structural properties of the network/ideological polarization, and not only rely on proxies measuring the attitude of users towards each others. (...) The authors should clarify why a measure of affective polarization (...) should depend on a structural metric.

    - We now refer to the "social distance component" instead of the "structural component" and have revised the section explaining this component. As discussed in the revised section, the political science literature considers social distance an important aspect of affective polarization. While social distance is typically measured through surveys, we argue that it can also be observed in social networks. Social distance reflects an avoidance behavior that manifests in the structure of social interactions.

Our revised Figure 5, which now displays the overall level of affective polarization alongside its separate components, illustrates how *both* affect and social distance (structure) matter. In the Twitter data, we first observe a rise in the affective component, which drops again as soon as the social distance component increases. In other words, users first act hostile towards others they disagree with and then stop interacting with them altogether. This underscores the importance of considering both affect and social distance when analyzing affective polarization.

2. Whether the two dimensions are treated as independent or correlated, the rationale behind multiplying these factors is not clear.

    - We added an explanation of the rationale in the Formulation section: Multiplying the two components enables us to preserve the sign flip from negative to positive. By doing so, we can capture two potential scenarios: a positive score (more disagreement, more hostility) and a negative score (more disagreement, less hostility). If we used a different operation, such as addition or subtraction, we would lose this ability to capture both directions of this relationship.

      While a negative value (more disagreement, less hostility) does not align with traditional definitions of affective polarization, we argue that a comprehensive measure should account for all possibilities. If such a counterintuitive scenario were to arise, our measure would draw attention to it, and it could be further investigated.

3. The section titled "Methods" should be renamed, as it overlaps with the later "Materials and Methods" section.

    - We have changed the section title to "Affective Polarization Measure" instead of "Methods".

4. It is unclear why the definition of the measure and the synthetic data analysis use Pearson correlation, whereas the other part of the analysis use Spearman correlation. If the correlation between hostility and disagreement is nonlinear (a finding I agree with), why is Pearson correlation used to define the measure?

    - We agree with the reviewer's comment and have revised the method section. We now use the Spearman rank correlation to define our proposed measure, noting that the Pearson correlation can be used instead if appropriate given the data. The results for the synthetic experiments (Figs. 2–4, Fig. S1) have also been updated.

5. The analysis of Twitter data is compelling; however, I suspect that the affective and structural dimensions of the measure are highly correlated. The authors should compare their measures with others and visualize the trends of the two components to provide additional insight.

    - We compare our measure to others in the synthetic experiments. To avoid overcomplicating the discussion of the Twitter results, we do not repeat this comparison here.

      Following a suggestion by Reviewer 1, we have updated Fig 5 to display our affective polarization measure alongside its two components. We find that the two components are negatively correlated (rank correlation coefficient of -0.3942): an initial rise in the affective component is followed by an increase in the social distance component, after which hostility declines while social

distance remains high. This suggests that users shift from direct hostility to avoidance.

6. The range of the proposed measure is not well-defined. This raises the question of whether the measure is suitable for comparing different networks or if it should be limited to tracking changes within the same network over time.

   - The range of our measure is $[-\infty, \infty]$ due to how we quantify social distance using the Generalized Euclidean (GE) distance. This distance metric has no upper bound because, for any given network G and opinion vector o, it is always possible to construct another network G' with an opinion vector o' that results in a higher GE score. For further details, please refer to the Supplementary Materials in [2].

     Importantly, both the Spearman rank correlation and the GE measure are scale-invariant. Therefore, our proposed affective polarization measure is also scale-invariant: it produces consistent values for networks with similar topologies, regardless of the number of nodes. Because of this property, our measure is well-suited for comparing different networks.

     That said, we caution against comparing networks that stem from fundamentally different data sources such as a Twitter network versus an offline friendship network. In such cases, variations in the measure may reflect differences in data generation processes rather than actual differences in affective polarization. However, this challenge is not unique to our measure but is a broader issue that applies to most comparative research designs.

Journal Requirements

1. Please ensure that your manuscript meets PLOS ONE's style requirements, including those for file naming.

   - We have used the provided LaTeX template – following the style requirements – to prepare the revised manuscript.

2. In your Methods section, please include additional information about your dataset and ensure that you have included a statement specifying whether the collection and analysis method complied with the terms and conditions for the source of the data.

   - We included the relevant statement in a subsection titled 'Data and Code Availability' under Materials and Methods.

3. Please note that ... your entire data will need to be made freely accessible if your manuscript is accepted for publication. If you are unable to adhere to our open data policy, please kindly revise your statement to explain your reasoning and we will seek the editor's input on an exemption.

   - As outlined in the new 'Code and Data Availability' subsection, we cannot make the Twitter data publicly available due to very strict limitations set out by the European Union's General Data Protection Regulation (GDPR). However, all code necessary to reproduce the synthetic data are made available.

     For peer review purposes, we have made the anonymized Twitter data available at https://osf.io/7w5ya/?view_only=54a7b7bce47b4f28ba52aaccb2b3e50d.

Please note that this link is only for peer review purposes and **not** to be publicly shared.

4. Please review your reference list to ensure that it is complete and correct. If you have cited papers that have been retracted, please include the rationale for doing so in the manuscript text, or remove these references and replace them with relevant current references. Any changes to the reference list should be mentioned in the rebuttal letter that accompanies your revised manuscript. ...

   - We carefully reviewed the reference list again and updated two items: [3] and [4]. Both references were preprints at the time of our initial manuscript submission and have since been published as journal articles.

References

1. Falkenberg M, Zollo F, Quattrociocchi W, Pfeffer J, Baronchelli A. Affective and interactional polarization align across countries; 2023. Available from: https://arxiv.org/abs/2311.18535.

2. Hohmann M, Devriendt K, Coscia M. Quantifying ideological polarization on a network using generalized Euclidean distance. Science Advances. 2023;9(9).

3. Fraxanet E, Pellert M, Schweighofer S, Gómez V, Garcia D. Unpacking polarization: Antagonism and alignment in signed networks of online interaction. PNAS Nexus. 2024;3(12).

4. Lerman K, Feldman D, He Z, Rao A. Affective polarization and dynamics of information spread in online networks. npj Complexity. 2024;1(1).
