# Supplementary material for: Estimating affective polarization on a social network
Source: PLoS One. 2025 Sep 24;20(9):e0328210. doi: 10.1371/journal.pone.0328210 (PMC12459850; doi:10.1371/journal.pone.0328210)
Supplement: Attachment — Submitted filename: response.pdf [file pone.0328210.s004.pdf]

Estimating affective polarization on a social network

Marilena Hohmann[1*], Michele Coscia[2]

1 Copenhagen Center for Social Data Science, University of Copenhagen, Copenhagen, Denmark
2 CS Department, IT University of Copenhagen, Copenhagen, Denmark

* marilena.hohmann@sodas.ku.dk

To the editors of PLOS ONE,

This document outlines our point-by-point rebuttal to the reviewers. For each point raised, we indicate where and how we modified the original text, or argue why we decided not to.

Reviewer 2

1. I still have concerns regarding the structural aspects analyzed in this work, now referred to as "social distance". While some studies consider this aspect a component or manifestation of affective polarization, others treat it as a distinct dimension of polarization. [...] In my view, the paper would benefit from being reframed as a comparison between these two dimensions of polarization. That said, I respect the authors' position on the matter and recognize that this remains an open question without a clear consensus in the field.

    - We think the reviewer has a solid point here. We have edited abstract, introduction, and discussion to improve our acknowledgment of this open question – and to allow the reader siding with the reviewer to still find usefulness in the method we develop by considering exclusively the affective component and relegating the social distance component as a distinct dimension.

References
